# Color signaling in conspicuous red sticklebacks: do ultraviolet signals surpass others?

**DOI:** 10.1186/1471-2148-8-189

**Published:** 2008-07-01

**Authors:** Ingolf P Rick, Theo CM Bakker

**Affiliations:** 1 Institut für Evolutionsbiologie und Ökologie, University of Bonn, An der Immenburg 1, D-53121 Bonn, Germany

## Abstract

**Background:**

The use of ultraviolet (UV) signals for communication tasks is widespread in vertebrates. For instance, there is a UV component to mate choice in several species. Nevertheless, it remains unclear how the signal value of the UV wave band compares to that of other regions of the animal's visible spectrum. We investigated the relative importance of UV signals compared with signals of longer wavelengths in the threespine stickleback (*Gasterosteus aculeatus*), a species using UV wavelengths in female and male mate choice as well as in shoaling behavior. In a choice experiment, female sticklebacks were simultaneously presented with four male visual appearances manipulated by optical filters. Each male lacked one wavelength range of the stickleback's visible spectrum corresponding to the spectral sensitivities of the four cone types. The resulting male appearances thus had no UV (UV-), no short-wave (SW-), no medium-wave (MW-) or no long-wave (LW-) body reflectance.

**Results:**

Males without UV wavelengths and long wavelengths ("red") were least preferred. In contrast, the removal of medium and most notably short wavelengths left male attractiveness to females rather unaffected. Using color metrics, the effects of the four optical filters on stickleback perception of three male body regions were illustrated as quantal catches calculated for the four single cones.

**Conclusion:**

The removal of UV light (UV-) considerably reduced visual attractiveness of courting males to female three-spined sticklebacks particularly in comparison to the removal of short-wave light (SW-). We thus report first experimental evidence that the UV wave band clearly outranks at least one other part of an animal's visible spectrum (SW-) in the context of communication. In addition, females were also less attracted to males presented without long wavelengths (LW-) which supports the traditionally considered strong influence of the red color component on stickleback mate choice. Overall, the removal of medium wavelengths (MW-) and especially short (SW-) left male attractiveness for females rather unaffected. Our work suggests that, in addition to long wavelengths ("red"), the UV wave band contains important information for visual mate choice in sticklebacks. Hence, the previously suggested exclusive role of the characteristic red nuptial coloration in visual interactions between reproductively active stickleback conspecifics may be overestimated with UV wavelengths playing a more important role than previously suggested.

## Background

UV visual signals are of recent interest in studies on social signaling, especially in the context of sexual selection. In several vertebrate species, there is a UV component to female mate-choice [1-7]. Nevertheless, until now, only a few experimental studies on birds demonstrated a condition-dependent expression of UV ornamentation [8,9] supporting a potential signaling value of UV coloration in sexual selection.

UV wavelengths are more strongly scattered in air and water than longer wavelengths [10] favoring communication over short distances with the signal being difficult to detect by more distant perceivers [11]. Thus, a proposed adaptive function underlying the development of UV signals is that these signals act as private communication channels [12], which enable communication between conspecifics without being detected by potential eavesdroppers, such as predators. However, experimental evidence for such a private visual communication channel is scarce. Cummings et al. [13] found in a behavioral study on a fish, the northern swordtail (*Xiphophorus nigrensis*), that male UV ornamentation significantly increased their attractiveness to females but not to a potential predator. Furthermore, using a visual model on color discrimination, Hastad et al. [14] investigated the conspicuousness of several European songbirds against typical visual backgrounds from either a conspecific's perspective or from a potential predatory bird's perspective. They found the songbird's plumage colors being better detectable by a conpecific than by a predator's eye due to differences in perceptual capabilities.

However, to gain more insight about some specialized role of UV signals in animal communication Stevens & Cuthill [15] conclude that it might be advantageous to focus on the relationship between the signal expression in the UV part and other spectral parts of animal coloration. This was done by Hunt et al. [16] who investigated the relative importance of UV light in a mate-choice context in zebra finches (*Taeniopyga guttata*). They did this by experimentally removing four different spectral parts of male plumage using optical filters that corresponded to the peak sensitivity of the four different single cone classes in zebra finches. Their results indicated that the removal of UV light had the least important effect on mate-choice decisions in this species, whereas the removal of longer wavelengths had the greatest influence. These findings corroborate the belief that the long-wavelength reflecting red beak coloration in zebra finch males is the most important component in visual mate-choice [17].

We studied the relative importance of UV wavelengths in the threespine stickleback (*Gasterosteus aculeatus*), a species showing a distinct sexual dichromatism with reproductively active males possessing conspicuous coloration in the human visible waverange mainly characterized by a red cheek and belly and a blue-green iris. The red nuptial coloration of male threespine sticklebacks is one of the earliest recognized and best known color signals in nature (reviewed in [18]) playing an important role in social communication such as female mate choice [19] and competition between males [20]. Accordingly, the red nuptial coloration in sticklebacks is positively correlated with physical condition, courtship intensity and female mating-preference [e.g., ref. [21]].

More recently, it was shown that threespine stickleback males also possess body regions with reflectance in the UV part of the spectrum [22] and a fourth UV sensitive cone-type was identified [23] in addition to three cone-types absorbing in the human-visible waverange [10]. Furthermore, UV communication is used in stickleback mate-choice that was recently demonstrated for female and male mating-preferences [6,7,24] as well as male-male interactions [25].

Given that UV signals take part in sexual interactions in the threespine stickleback, we investigate in the present study on female mate-choice, how these signals compare with signals of longer wavelengths, especially the characteristic red nuptial coloration. We tested female response behavior for four male appearances each manipulated by optical filters that excluded different spectral wave bands from the stickleback's perceptual range. Removed spectral regions corresponded closely to the sensitivity of the four single cone classes in the stickleback retina. Our experiment was based on the assumption that, if a particular wave band is of special importance in female mating-preference its removal will lead to a considerable decrease in female preference compared to rather irrelevant wave bands. In contrast to this, the absence of a less important wave band should not greatly reduce male attractiveness to females.

In addition, we used color metrics for calculating the predicted effects of the four optical filters on the perception of three male body regions from the female's perspective and related these effects to the choice behavior females showed during the experimental trials.

## Methods

### Experimental subjects

Samples of adult sticklebacks were collected in April 2006 from a shallow pond near Euskirchen, Germany (50°38'N, 6°47'E). In the laboratory, fish were maintained in outside stock tanks (volume 700 litres; temperature 15°C with a tap-water flow rate of 3 litres/min and air ventilation). Individuals were fed to excess once daily with frozen chironomid larvae. After two weeks, males that showed the typical red nuptial coloration were moved individually into aquaria (30 × 20 × 20 cm, 12 litres) in the laboratory. Each aquarium was equipped with a petri dish filled with fine gravel to provide males with a nesting site and 150 threads, each 4 cm long, of green cotton twine (100% cotton, Patricia, Germany), as nesting material. The fish were maintained at 17° ± 2°C under a 16:8 light:dark illumination cycle provided by fluorescent tubes (True Light, Aura Light, Germany, Natural Daylight 5500, 36 W, 1200 mm). The tubes produced a proportion of UV similar to natural skylight and were suspended 20 cm above the tanks. To induce nest-building behavior, we stimulated each male once a day for 10 minutes by presenting a ripe female in a 500-ml jar in front of the holding aquarium. Females were also transferred into the laboratory and placed in group-tanks (45 litres) with 10 individuals each. Single males and females were held under similar conditions. All fish were fed *ad libitum *with frozen chironomid larvae once daily.

### Experimental set-up

**Figure 1:**
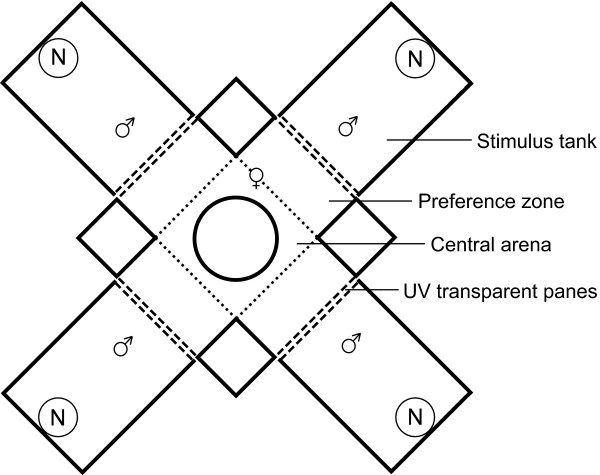
**Plan view of the choice apparatus**. The central arena for the choosing female consisted of a central neutral zone with an opaque plastic cylinder in the middle and four preference zones, which are separated from the central zone by dotted lines. The four tanks for the stimulus males contained the males' nests (N) and were placed perpendicular to the choice chambers. Solid lines represent opaque plastic walls whereas dashed lines represent UV transparent Plexiglas panes. Optical filters were placed horizontally over the four stimulus tanks.

**Figure 2:**
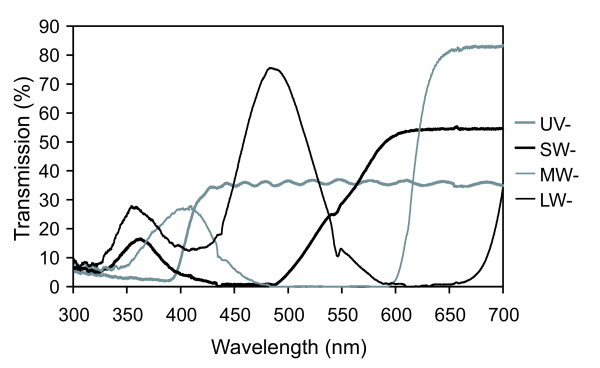
**Transmission of treatment filters**. Transmission spectra for the four optical filters used in the stimulus and control experiment (UV-blocking (UV-), short-wave-blocking (SW-), medium-wave-blocking (MW-) and long-wave-blocking (LW-)). Spectra were measured with an Avantes AVS-USB2000 spectrometer connected to an Avantes DH-2000 deuterium-halogen light source. Transmission was determined by attaching the reflection probe at a 90° angle to the measured filter located on a 98% white standard.

Mate-choice trials were conducted using a cross-shaped experimental design (Figure 1), which was basically similar to the one used in studies on zebra finch visual preferences [1,16]. One choosing female was placed in the central arena, whereas four males were placed in individual stimulus tanks (30 × 20 × 20 cm, 12 litres), each positioned with its transparent side connected to the arena. The water level in the tanks and arena was 14 cm. The arena had chambers in front of the four stimulus tanks (20 × 12 cm), which were declared as preference zones. The panes located between the sexes consisted of UV-transparent Plexiglas (GS2458, Röhm, Germany) whereas the remaining walls consisted of opaque, grey plastic partitions surrounding the whole set-up. In order to prevent interactions between males from opposite stimulus tanks that could have influenced the female-male interaction, an opaque, grey plastic cylinder (13.5 cm × 20 cm) was placed centrally in the choice tank. The set-up was illuminated by four fluorescent tubes (True Light, Aura Light, Germany, Natural Daylight 5500, 36 W, 1200 mm) placed 20 cm above the outer walls of the stimulus tanks and tilted in a 30° angle towards the centre of the set-up. The tubes were arranged in a square that was horizontally rotated in a 45° angle to the central arena. This arrangement produced equal lighting conditions for the four stimulus tanks with illumination slightly attenuating towards the central area of the set-up. The central choice arena was illuminated by unfiltered light whereas the stimulus tanks were covered with four different color filters. These filters manipulated the male's appearance to the choosing female [see also ref. [16]] by removing discrete wave bands which coincided with the spectral sensitivity of the four stickleback cone types [see ref. [23]]. The filters are referred to as UV-blocking (UV-), short-wave-blocking (SW-), medium-wave-blocking (MW-) and long-wave-blocking (LW-) (Lee Filter No. 229, Rosco Supergel Filters 14, 339 and 73, respectively) (Figure 2). Attenuation of the filters was balanced by using multiple layers of filter material. The exact ratios of quantal flux (the total amount of light transmitted between 300–700 nm) for the four treatments (UV-:SW-:MW-:LW-) were 1.19:1.13:1.15:1.00. Opaque partitions (20 × 20 cm) were placed between the stimulus tanks and the central arena which were lifted during the test phase up to a height of 14 cm. These partitions reduced the amount of light passing through the filters on top of the stimulus tanks and through the UV transparent panes into the central choice arena which otherwise could have altered female appearance for the different males. On the other hand, the opaque partitions combined with the opaque plastic cylinder located in the centre of the choice arena largely prevented that fullspectrum light could pass from the choice arena into the stimulus tanks. Hence, male stimuli appeared nearly entirely under the relevant filtered light instead of a mixture of fullspectrum and filtered light.

### Experimental procedure

Twelve males and females were used for the stimulus experiment. The 12 females were divided into three groups of four fish. Each group was randomly assigned a group of four males so that the females within each group viewed the same four males during the experimental trials. By changing filters between trials following a Latin square design each female viewed a different male-filter set.

Before being used in experimental trials males had to complete their nests. The four males within each group were matched for body length (± 1 mm) and weight (± 100 mg). Males were transferred to the experimental set-up one day before the first trial and stayed in their stimulus compartments until all four trials had been finished. Trials were conducted on four consecutive days. During this period each male was fed with frozen chironomid larvae *ad libitum *and air ventilation was supplied. When a ripe female was transferred to the experimental set-up a 1 h acclimatization phase started with the opaque partitions placed between the males and the female. In the following observation phase the opaque partitions were lifted which enabled the female to observe the four stimulus males. This phase lasted until the female had frequented all four preference-chambers. Then the 10-minute test phase started in which female behavior was recorded. After that, the opaque partitions were replaced and the female was removed from the experimental set-up. This procedure was repeated on the following day with a different female and the same males under changed filters. The water of the choice arena was replaced after each trial. All trials were conducted in normal daylight hours. Only females that spawned within 24 h after the end of the experimental trials were regarded as reproductively active and thus included in analyses.

We filmed and recorded each trial from above with a webcam connected to a laptop. We measured the time that the female (entire body) spent in the four preference chambers in front of the stimulus tanks during the test phase. Filming from above did not allow for recording further behavioral patterns indicating female choice, such as the characteristic head-up display. However, association time is an approved measure for mating preference in sticklebacks [26-28].

After finishing the stimulus experiment, a supplementary control experiment was conducted with eight additional females to test for general preferences for the four light environments created by the optical filters. Experimental trials were performed analogous to the stimulus experiment except for leaving out the male stimuli. Films for the stimulus and the control trials were analysed blind, that is, without knowledge of trial type and filter positions by covering the colored filters on the computer monitor.

All data on female behavior were normally distributed. We analyzed the relative amount of time females spent near the four male appearances in the stimulus experiment with a repeated-measures analysis of variance in SPSS. The relative amount of time females spent near the four light environments in the control experiment was analyzed using an analysis of variance.

### Color metrics

To discover potential effects of color perception on female behavior in the stimulus experiment we used color metrics [29] as a first approach to examine signal perception. We asked how differences in male reflectance between the color treatments might be detected as differences in the proportional response of stickleback visual pigments. Therefore, we took mean spectral reflectance data recorded from two body regions (cheek, abdominal region) of 14 males from another study [25] and additionally, using the same measurement protocol, assessed mean spectral reflectance from one body region (ventral spine) of eight further males. Males used in the present study and males used for reflectance analyses derived from the same study population.

Our subsequent calculations were based on cone absorption spectra presented by Rowe et al. [30]. Following the equation of Vorobyev et al. [31], absolute quantal catch rates of the four cone receptor types (UV, S, M, L) were calculated by multiplying the reflectance spectrum of each body region by the spectral sensitivity of the cones and the irradiance spectrum of the fluorescent tubes summed across wavelengths between 300 and 700 nm. Furthermore, due to a low water level and short signaling distances between individuals based on our experimental set-up we did not include absorption and scatter properties by water in our computations. We also did not account for ocular media transmission in our analysis since lens absorbance in sticklebacks from our study population can be neglected for the spectral range considered in this study [I. P. Rick, unpublished data].

In our analysis it is important to consider the photoreceptor's adaption state when viewing an object under different light conditions [31]. Thus, following Hunt et al. [16] we assumed that the stickleback cones did not adapt to each particular filter treatment but rather were generally adapted to the stimulus tank background illuminated by unfiltered light as perceived in the central choice arena. This background consisted of abrased, grey plastic partitions offering nearly even broadband reflectance across the UV and human visible waveband and was defined to be located at the color space centre by providing equal stimulation of all cones [32]. Hence, to model adaptation to the stimulus tank background in fullspectrum light the corresponding quantal catch rates were fixed, which means that all cones were equally stimulated. Accordingly, cone excitations for the three body regions under the different treatment filter were set relative to those for the stimulus tank background. We next calculated relative quantal catches by dividing excitation of each cone by the sum of excitation for all four cone classes (*e.g. *UV = [UV + S + M + L]). The determined relative quantal catches express the coordinates of the perceived color in the color space [29].

Since detailed psychophysical data on stickleback perception is lacking our calculations are merely approximative with regard to the colors actually perceived by the female's eye. However, our model should be suitable for simply illustrating the differences in cone stimulation between the four filter treatments, which generate visual male stimuli that considerably differ in spectral information.

## Results

### Experimental set-up

**Figure 3:**
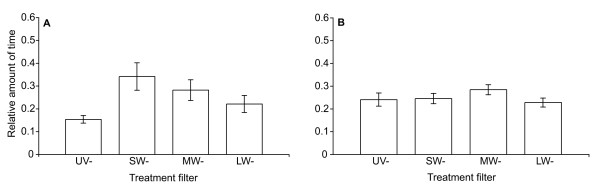
**Female filter preference**. (A) Mean relative time ± SEM spent by 12 females within the preference zones in front of males under the ultraviolet-blocking (UV-), short-wave-blocking (SW-), medium-wave-blocking (MW-) and long-wave-blocking (LW-) treatment filters during the 10 min test phase of the stimulus experiment. (B) Mean relative time ± SEM spent by eight females within the preference zones in front of empty tanks under the UV-, SW-, MW- and LW- treatment filters during the 10 min test phase of the control experiment.

All 12 females used in the stimulus experiment had released eggs within 24 h after the end of the experimental trials and were thus included in the analysis. In the stimulus trials, females significantly discriminated between the four filter treatments (ANOVA: F_3.33 _= 3.600, P = 0.026, Figure 3a). Treatment differences were examined with a series of orthogonal contrasts. Three simple models for preference ranking order fitted the data well. The best-fitting model was a linearly increasing order of preference UV- < LW- < MW- < SW- (t = 3.268, P = 0.002) followed by the preference ranking UV- < LW- = MW- < SW- (t = 3,107, P = 0.003). The preference ranking UV- = LW- < MW- = SW- also gave a good fit to the data (t = 2.914, P = 0.006). Overall, female sticklebacks showed the lowest preference for males presented under filters removing the UV (UV-). Removing long wavelengths (LW-) had a similar strong effect, whereas the absence of medium (MW-) and in particular short wavelengths (SW-) had the least effect on female preference.

All eight females in the control experiment had released eggs within 24 h after the end of the experimental trials and were thus included in the analysis. Without stimulus males, females showed no significant discrimination between the four filter treatments (ANOVA: F_3,21 _= 1.067, P = 0.379, Fig 3b). Female preference in the stimulus trials was thus likely based on the perception of differences between male visual appearances instead of differences between the ambient light conditions in the stimulus tanks.

### Color metrics

**Figure 4:**
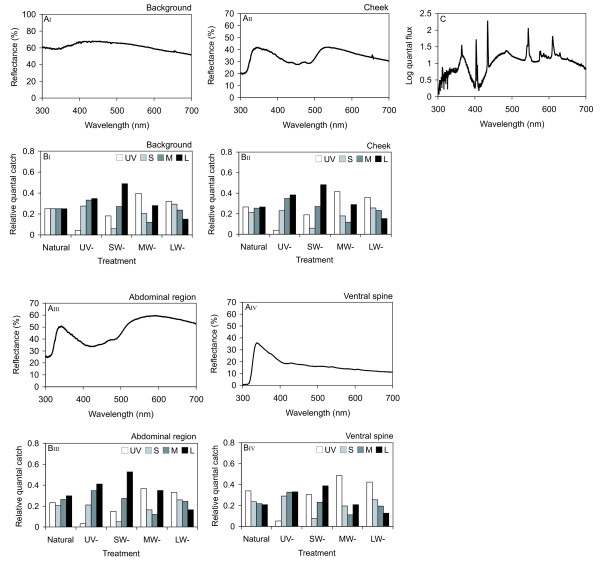
**Male body reflectance, quantal catches for the different light treatments and irradiance of unfiltered light**. Mean reflectance of (A_I_) the background, (A_II_) cheek region, (A_III_) the abdominal region and (A_IV_) the ventral spine of male sticklebacks. (B_I_-B_IV_) Relative quantal catches calculated for the four stickleback cone classes (UV, S, M, L) affected by the reflectance of the experimental background and each male body region under fullspectrum illumination (Natural) and under the four treatment filters (UV-, SW-, MW-, LW-). Quantal catches are calculated relative to the excitation of the four cone types by the stimulus background under unfiltered light (C) Relative irradiance (log quantal flux) of the True Light tubes used in the experiment and visual modelling. Irradiance was measured with an Avantes AVS-USB2000 connected to an Avantes CC-UV/VIS cosine corrector located in the stimulus tank centre with filters removed. Irradiance calibration was performed versus an Avantes NIST traceable irradiance application standard.

Reflectance spectra from the experimental background, cheek region, abdominal region, and ventral spine of stickleback males are shown in Figure 4A_I_-A_IV_. The correspondent quantal catches by each of the four single cones under natural illumination (Truelight tubes) and each of the four light treatments are given in Figure 4B_I_-B_IV_. As an example, the cheek reflectance pattern was bimodal peaking in the ultraviolet and in the human visible wave band (Figure 4A_II_), and under natural illumination the UV and L cones are stimulated stronger than the M and still more stronger than the S cone (Figure 4B_II_). Relative quantal catches for the cheek under the filtered irradiance treatments display how one respective cone signal is reduced (e.g. lower quantal catches in the UV cone when UV wavelengths are blocked (UV-) as well as lower quantal catches in the LW cone when long wavelengths are blocked (LW-)). The gonadal region also showed a bimodal reflectance spectrum similar to the cheek (Figure 4A_III_), which results in similar cone excitations (Figure 4B_III_). In contrast, the ventral spine revealed a unimodal spectrum with a single reflectance peak in the UV region (Figure 4A_IV_). Hence, for the ventral spine quantal catch ratios especially in the L cone are lower compared to the other two sample regions for all light treatments (Figure 4B_IV_).

## Discussion

Our study provides the first evidence that UV wavelengths are of higher relative importance than at least one other part (SW-) of an animal's visible spectrum in a visual communication context. More in detail, the removal of UV light (UV-) considerably reduced visual attractiveness of courting males to female threespine sticklebacks in comparison to light of short wavelengths (SW-). The male appearance without long wavelengths (LW-) also was less attractive than that without short wavelengths (SW-), although the effect was slightly weaker than for the UV- treatment. Overall, the absence of short (SW-) and medium (MW-) had the least strong effect probably even leaving male attractiveness rather unaffected.

The treatment filters produced different light environments for the stimulus males and thus may have influenced male behavior, which again may have affected female choice behavior. We could not check this since transmission of the treatment filters was too low for recording male behavior. However, our experimental design with horizontally positioned filters ensured that the test female did not appear different to each male which otherwise might have resulted in distinct differences in male behavior.

Furthermore, the treatment filters notably changed the ambient light conditions in the stimulus tanks and thus may have caused differences in female preference independent from the stimulus male appearance. But, when given the choice between empty stimulus tanks females did not significantly prefer a particular light habitat suggesting that the preference found in the stimulus trials was rather influenced by the stimulus male's appearance alone.

When comparing female filter preference in the stimulus trials with that in the control trials the difference in the relative female preference for the UV- males and SW- males becomes more pronounced whereas the preference for the MW- and LW- male filter combination is similar for both trial types. However, when considering only the stimulus experiment, one can conclude that in addition to the absence of UV (UV-) the absence of long wavelengths (LW-) caused males to be less attractive to females than under the MW- and LW- treatment filters.

Other than the strong effect of removing UV light (UV-), the low female preference for males under conditions lacking long wavelengths (LW-) is less surprising considering the importance of male red nuptial coloration in stickleback mate-choice [33]. Correspondingly, Milinski and Bakker [21] found that females when presented with courting males under green light filtering out longer wavelengths were unable to assess differences in male red coloration.

Our experimental approach is based on the assumption that sticklebacks possess a tetrachromatic visual system. Although this is a valid assumption, it is unknown whether the UV cone class in sticklebacks participates in tetrachromatic vision since the availability of UV cones in the retina does not automatically mean that these cones contribute to color vision. Performing color mixture experiments in the goldfish (*Carassius auratus*), Neumeyer [34] demonstrated that the UV cone class is involved in color vision in this species. For the threespine stickleback, a similar behavioral approach as well as better knowledge of the neuronal pathways used in visual processing would be helpful to clarify whether color vision is based on all four cone types.

As mentioned before, Hunt et al. [16] found that female mate-choice in zebra finches was less affected by the removal of UV wavelengths than by the removal of long-wave light. This is in accordance to the fact that red colored plumage plays an important role in visual communication in this species, whereas UV reflectance is absent in these plumage regions. In comparison, long-wave reflecting body regions in the threespine stickleback (e.g. the red cheek) reveal a double-peaked reflectance spectrum including an additional amount of UV reflectance presumably produced by broadband reflecting structural coloration in combination with absorption of carotenoids in the 400–500-nm range. The bimodal reflectance pattern found in the present study is also echoed in the relative cone catches estimated for the different filter treatments in our experiment which are consistently higher for the UV cone compared to the zebra finch cone catches given by Hunt et al. [16].

Assuming that the relative importance of different parts of an animal's visual spectrum corresponds to their relevance as a color signal used in visual communication, our results suggest that the UV wave band in addition to long wavelengths ("red") may provide crucial information for visual mate-choice in sticklebacks. Nonetheless, to our knowledge, it is virtually unknown whether UV ornamentation in male sticklebacks signals some aspect of male quality used by females. In male eastern bluebirds (*Sialia sialis*), Siefferman & Hill [8] found that males with a higher expression of UV ornamentation were better competitors and fledged more offspring. Furthermore, in blue tits (*Parus caeruleus*) males with a higher UV chroma enjoyed fitness advantages because their females produced more male offspring and showed higher parental effort [35,36]. In stickleback males, the UV contrast of the abdominal region was positively correlated with body condition [22] suggesting a potential signaling role in sexual selection. However, further work addressing a potential condition-dependent expression of UV signals is necessary to solve whether UV-reflecting structural coloration gives females reliable information about male quality as it was demonstrated for the carotenoid-based red component of male nuptial coloration [e.g., ref. [21]].

Beyond, especially for the red cheek coloration an interaction between structural and pigmentary color components is possible when, for example, an altered deposition of carotenoids leads to differences in UV reflectance. Mougeot et al. [37] found a negative relationship between the expression of red and UV comb coloration in a bird, the red grouse (*Lagopus lagopus scoticus*), and hypothesized that UV reflectance is masked by carotenoid pigmentation. One can assume a similar interaction between UV and red color components of male sticklebacks that may be important for female mate assessment since females in our experiment were more attracted to males presented under the SW- and, even though to a lesser extent, MW- treatment filters, both enabling the transmission of UV and long-wave light. This is also reflected in the particular cone catch values obtained for the red cheek region under SW- and MW- conditions where a simultaneous excitation of both, the UV and L cones is given. In comparison, females were less attracted to males presented under UV- conditions with low excitation values in the UV cone and high values in the L cone and vice versa under LW- conditions.

However, to get more insights into how the overall visual appearance of stickleback males affects female mating decisions, future research, whilst taking UV wavelengths into account, should integrate the role of visual contrast between different body regions as well as between body regions and the background. For example, our study did not address the blue-colored iris which is a further conspicuous component of male nuptial coloration and together with the red throat and dark flanks seen as part of a high-contrast mosaic pattern [21]. In this regard, Rush et al. [38], while omitting wavelengths lower than 350 nm in their analysis, demonstrated that in male sticklebacks the visual contrast between the blue eye and red throat is elevated under social stimulation causing an increase in overall conspicuousness.

## Conclusion

To conclude, by systematically investigating the role of different parts of the visual spectrum, our work suggests that the exclusive role of the red nuptial coloration in stickleback mate-choice may be overstated with UV wavelengths having a more important function than previously assumed. More generally, the relative importance of UV light in visual signaling found in our study contradicts previous investigations on other species where UV was classified to be a rather unimportant component in animal communication [16,39]. Future work thus should consider species-specific and also population-specific attributes of visual communication including more detailed information about signal properties, the ambient light environment in the natural habitat and the sensory experience of a potential receiver.

## Authors' contributions

IPR devised the study, participated in the conception and planning of the experiments, carried out the experiments, collected and analysed the behavioral data, collected and analysed the color metrics, performed all statistical analyses, participated in the discussion of the results and wrote the manuscript. TCMB participated in the conception and planning of the experiments, contributed to discussion of results and wrote the manuscript. All authors read and approved the final manuscript.
